# Factors Predicting Client Re-Enrollment in Tobacco Cessation Services in a State Quitline

**DOI:** 10.5888/pcd15.180144

**Published:** 2018-10-18

**Authors:** Uma S. Nair, Benjamin R. Brady, Patrick A. O’Connor, Melanie L. Bell

**Affiliations:** 1 Department of Health Promotion Sciences, Mel and Enid Zuckerman College of Public Health, University of Arizona, Tucson, Arizona; 2 Department of Epidemiology and Biostatistics, Mel and Enid Zuckerman College of Public Health, University of Arizona, Tucson, Arizona

## Abstract

**Introduction:**

Quitlines are an integral part of tobacco treatment programs and reach groups of smokers who have a wide range of barriers to cessation. Although tobacco dependence is chronic and relapsing, little research exists on factors that predict the likelihood of clients re-engaging and reconnecting with quitlines for treatment. The objective of this study was to describe factors that predict the re-enrollment of clients in Arizona’s state quitline.

**Methods:**

This was a retrospective analysis of data collected from clients (N = 49,284) enrolled in the Arizona Smokers’ Helpline from January 2011 through June 2016. We used logistic regression to analyze predictors of re-enrollment in services after controlling for theoretically relevant baseline variables (eg, nicotine dependence, smokers in the home) and follow-up variables (eg, program use, quit outcome).

**Results:**

Compared with clients who reported being quit after their first enrollment, clients who reported not being quit were almost 3 times as likely to re-enroll (odds ratio = 2.89; 95% confidence interval, 2.54–3.30). Other predictors were having a chronic condition or a mental health condition, greater nicotine dependence, and lower levels of social support. Women and clients not having other smokers in the home were more likely to re-enroll than were men and clients not living with other smokers.

**Conclusion:**

Understanding baseline and in-program factors that predict client-initiated re-enrollment can help quitlines tailor strategies to proactively re-engage clients who may have difficulty maintaining long-term abstinence.

## Introduction

Smoking is the leading preventable cause of death and disease (1). In the United States, almost 70% of people who smoke report an intention to quit and just over half attempt to do so (2). Tobacco dependence is chronic and relapsing: 60% to 90% of smokers attempting to quit relapse within 12 months (3). After relapse, many are motivated to try again, and the average smoker makes approximately 30 lifetime quit attempts (4). Thus, when smokers participating in cessation treatment programs are not initially successful, many continue to be interested in seeking cessation treatment (5) and can benefit from re-engagement in services (6–8).

Quitlines are an integral component of comprehensive tobacco control strategies (9), and evidence points to the effectiveness of telephone-based interventions on smoking cessation (10). Quitlines may be missing an opportunity to reconnect and re-engage former clients who may be at high risk for relapse or would benefit from additional cessation services. Although reconnecting and re-engaging former clients is feasible and effective for quitlines (11,12), a study showed that only 12 of 62 quitlines (19%) reported recontacting relapsed smokers for re-enrollment (13). Understanding baseline and program factors that predict client self-initiated re-enrollment in quitlines can inform the ability of quitlines to tailor outreach and services for clients most likely to re-enroll. Quitline re-enrollment is an unexplored area of research. The primary objective of this study was to describe factors that may predict client re-enrollment in Arizona Smokers’ Helpline (ASHLine), Arizona’s state quitline. A secondary objective was to explore differences between clients who re-enrolled and those who did not among those who reported smoking at 7-month follow-up.

## Methods

In this retrospective cohort study, we collected data from clients enrolled in the ASHLine program from January 1, 2011, through June 26, 2016. All assessments were conducted via telephone by trained survey staff using standardized protocols. The study used de-identified client data and was reviewed and deemed exempt by the University of Arizona’s institutional review board. Per protocol, all enrolled clients were assigned a tobacco cessation coach within 24 to 48 hours of enrollment. Clients were trained in evidence-based skills to develop urge and stimulus control strategies and received guidance on tobacco cessation and relapse prevention. In conjunction with behavioral coaching, eligible clients also received up to 4 weeks of nicotine replacement therapy (NRT, eg, gum, patch, lozenge).

### Measures

The primary outcome was re-enrollment in ASHLine, which was categorized as one-time–only enrollment or re-enrollment. Re-enrollment was defined as enrolling in services 2 or more times during the study period.

Predictor variables were the following prespecified baseline variables: age (as a continuous variable in years), sex (male or female), mode of entry in the program (provider-referred or self-referred), insurance type (Medicaid, private, uninsured), social support (poor, fair, good, very good, or excellent), other smokers in the home (yes or no), nicotine dependence (assessed by the Fagerström Test for Nicotine Dependence [14], the scores for which range from 0 to 10, with larger values indicating greater dependence); presence of a chronic condition or mental health condition (yes or no); and confidence to quit for 24 hours (extremely confident, very confident, confident or somewhat confident, or not confident). Data on follow-up variables were collected by telephone survey at 7 months after the first enrollment only. Follow-up variables were program use (use of NRT and number of telephone counseling sessions) and quit outcome. Quit outcome was measured by the response to the question “Have you used tobacco products in the last 30 days?” Thirty-day abstinence was recorded as yes or no.

### Statistical analysis

We calculated descriptive statistics for baseline variables and 7-month follow-up predictor variables. We examined differences in variables between the 2 groups (one-time–only and re-enrollment) by using χ^2^ tests and *t* tests. Logistic regression was used to examine predictors of re-enrollment and to estimate the association between baseline factors and re-enrollment (model 1). Our rationale for using a baseline-variable–only model (model 1) was to determine whether we could achieve model fit and prediction similar to that achieved in a model that included postbaseline variables. In model 1, because logistic regressions use complete case observations, we excluded from analysis data on clients for whom we were missing baseline information. Model 2 included all covariates from model 1 plus quit status at 7-month follow-up (abstinent in previous 30 days or not abstinent), number of telephone counseling sessions before 7-month follow-up (0–4 or ≥5), and use of NRT after baseline (yes or no). Including the 3 postbaseline covariates decreased the sample size from 34,552 observations in model 1 to 12,120 observations in model 2 because of missing data (Figure). We estimated odds ratios (ORs), 95% confidence intervals (CIs), and *P* values. The assumption of linearity between the logit of the outcome and each predictor was tested by using a loess curve plotted on a scatterplot. Any continuous variables violating this assumption were categorized. To assess model fit, we used the Hosmer–Lemeshow test and the *C* index. *C* index values range from 0.5 to 1.0; a *C* index of 0.5 indicates a model with no predictive ability. We used the likelihood ratio test to assess any difference between model 1 and model 2. All values for all variables were based on clients’ first enrollment in ASHLine.

**Figure:**
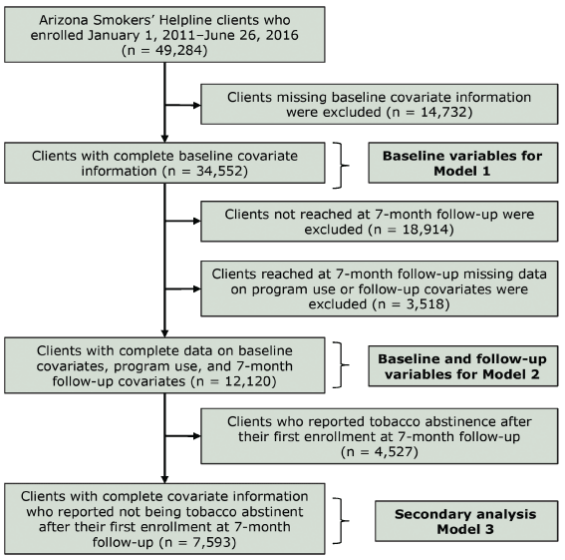
Arizona Smokers’ Helpline clients included in the analysis to predict client re-enrollment, January 1, 2011, through June 26, 2016.

We performed a secondary analysis to explore differences between clients who re-enrolled and those who enrolled one time only among clients who reported using tobacco in the previous 30 days at 7-month follow-up after their first enrollment (model 3). Model 3 assumptions were checked, and fit was assessed by using the same methods used for model 2. As in model 2, we excluded from analysis data on clients for whom we were missing baseline or follow-up information. All analyses were performed by using SAS version 9.4 (SAS Institute Inc).

## Results

During the study period, ASHLine enrolled 49,284 unique clients, of whom 4,740 (9.6%) re-enrolled at least one additional time (Table 1). After their first enrollment, we reached 12,120 clients for their 7-month follow-up (Figure). Of the 62.6% (n = 7,593), not reporting abstinence at follow-up, only 18.6% (n = 1,414) re-enrolled into services.

**Table 1:** Characteristics of Clients Enrolled in Arizona Smokers’ Helpline (N = 49,284), January 1, 2011–June 26, 2016^a^

Variable	One-Time–Only Enrollment	Re-Enrollment	*P* Value^b^
**Baseline^c^**			
**No. of respondents**	44,417	4,739	—
**Age, mean (SD), y**	48.8 (14.2)	51.2 (12.6)	<.001
**Sex**			
Female	24,953 (56.5)	2,895 (61.6)	<.001
Male	19,177 (43.5)	1,807 (38.4)	
Missing data	414	38	—
**Mode of entry into program**			
Self-referred	33,078 (74.3)	3,705 (78.2)	<.001
Provider-referred	11,466 (25.7)	1,035 (21.8)	
Missing data	0	0	—
**Insurance type**			
Uninsured	12,898 (29.1)	1,296 (27.5)	.01
Medicaid	10,062 (22.7)	1,022 (21.7)	
Private insurance	21,361 (48.2)	2,403 (50.9)	
Missing data	223	20	—
**Social support **			
Poor or fair	8,240 (22.3)	995 (25.6)	<.001
Good, very good, or excellent	28,676 (77.7)	2,899 (74.4)	
Missing data	7,628	846	—
**Other smokers in the home**			
Yes	17,988 (49.7)	1,770 (46.3)	<.001
No	18,228 (50.3)	2,052 (53.7)	
Missing data	8,328	918	—
**Score for Fagerström Test for Nicotine Dependence (range, 0–10), mean (SD)^d^**	4.7 (2.3)	5.0 (2.3)	<.001
**Mental health condition**			
Yes	15,977 (37.5)	2,117 (47.1)	<.001
No	26,593 (62.5)	2,375 (52.9)	
Missing data	1,974	248	—
**Chronic health condition**			
Yes	24,014 (55.8)	2,829 (62.3)	<.001
No	18,989 (44.2)	1,713 (37.7)	
Missing data	1,541	198	—
**Confidence to quit for 24 hours**			
Somewhat or not likely	5,242 (14.2)	545 (14.0)	.80
Very or extremely likely	31,730 (85.8)	3,339 (86.0)	
Missing data	7,572	856	—
**Intention to quit in next 30 days**			
No or don’t know	1,313 (3.5)	150 (3.8)	.39
Yes, I have already quit	36,284 (96.5)	3,841 (96.2)	
Missing data	6,947	749	—
**No. of cigarettes per day, mean (SD)^e^**	17.4 (10.1)	18.0 (10.5)	.01
**Education**			
<High school	20,101 (46.5)	2,044 (44.6)	.01
≥Some college	23,103 (53.5)	2,544 (55.4)	
Missing data	1,340	152	—
**In Program**			
**Program exit reason**			
Completed program	4,941 (11.9)	448 (9.9)	<.001
Quit and no longer wants service	1,537 (3.7)	131 (2.9)	
Not quit, relapsed, or unable to reach	35,051 (84.4)	3,960 (87.2)	
Missing data	3,015	201	—
**No. of telephone counseling sessions before 7-month follow-up**			
0–4	33,365 (74.9)	3,406 (71.9)	<.001
≥5	11,179 (25.1)	1,334 (28.1)	
Missing data	0	0	—
**No. of days in program, mean (SD)^f^**	63.0 (58.3)	68.3 (68.6)	<.001
**7-Month Follow-Up^g^**			
**30-day cessation**			
Quit	7,348 (39.4)	555 (19.0)	<.001
Not quit	11,310 (60.6)	2,372 (81.0)	
Missing data	7	0	—
**Use of nicotine replacement therapy **			
Yes	10,466 (72.4)	1,815 (73.9)	.13
No	3,981 (27.6)	640 (26.1)	
Missing data	4,218	472	—
**Home smoking ban at 7-month follow-up**			
Yes	12,718 (79.3)	1,959 (72.5)	<.001
No	3,315 (20.7)	742 (27.5)	
Missing data	2,632	226	—

Abbreviations: SD, standard deviation.

a Categorical values are presented as number (percentage) and continuous variables as mean (SD). Re-enrollment was defined as enrolling ≥2 times. All values were based on clients’ first enrollment in the quitline. Not all clients answered all questions, so n’s vary by question.

b *P* values for categorical variables were found by using χ^2^ tests and *P* values for continuous variables were found by using *t* tests.

c At baseline, n = 44,544 for clients who enrolled one time only and n = 4,740 for clients who re-enrolled.

d n = 35,403 for one-time enrollment; n = 3,774 for re-enrollment.

e n = 35,574 for one-time enrollment; n = 3,820 for re-enrollment.

f n = 43,138 for one-time enrollment; n = 4,677 for re-enrollment.

g At 7-month follow-up, n = 18,665 for clients who enrolled one time only and n = 2,927 for clients who re-enrolled.

Compared with clients who enrolled one time only, re-enrolled clients were significantly more likely to have a mental health condition (47.1% vs 37.5%, *P* < .001) or a chronic health condition (62.3% vs 55.8%, *P* < .001) and were less likely to be abstinent in the previous 30 days at 7-month follow-up (19.0% vs 39.4%, *P* < .001). Re-enrolled clients, compared with one-time–only clients, were also significantly more likely to be women, to be older, to have higher nicotine dependence, to report having lower levels of social support, to have self-referred into the program, and to have received 5 or more telephone counseling sessions (Table 1). We found no difference in use of NRT between one-time–only clients and re-enrolled clients.

The likelihood ratio test comparing model 1 and model 2 showed that model 2 fit the data better than model 1 (*P* < .001) (Table 2). In model 2, clients who were not abstinent for 30 days at 7-month follow-up were almost 3 times as likely to re-enroll in services compared with those who were abstinent (OR = 2.89; 95% CI, 2.54–3.30). Having a mental health condition (OR = 1.29, 95% CI, 1.15–1.44), having a chronic health condition (OR = 1.14; 95% CI, 1.02–1.28), or using smoking-cessation medication while in the program (OR = 1.14; 95% CI, 1.00–1.29) also increased the odds of re-enrollment. Factors that decreased the odds of re-enrollment were being male (OR = 0.77; 95% CI, 0.69–0.86) and living with other smokers in the home (OR = 0.83; 95% CI, 0.74–0.92). Finally, having some form of insurance coverage (Medicaid [OR = 0.68; 95% CI, 0.58–0.80], private insurance [OR = 0.87; 95% CI, 0.77–0.99]) significantly reduced the odds of re-enrollment, compared with having no insurance.

**Table 2:** Likelihood of Re-Enrollment (Enrolling ≥2 Times) in Arizona Smokers’ Helpline, Compared With One-Time–Only Enrollment, January 1, 2011–June 26, 2016

Variable	Model 1 (n = 34,552)^a^		Model 2 (n = 12,120)^b^	
	OR (95% CI)	*P* Value	OR (95% CI)	*P* Value
**Baseline**				
**Age, per 10-year increase**	1.12 (1.09–1.15)	<.001	1.04 (1.00–1.09)	.06
**Sex**				
Female	1 [Reference]		1 [Reference]	
Male	0.83 (0.77–0.90)	<.001	0.77 (0.69–0.86)	<.001
**Mode of entry into program**				
Self-referral	1 [Reference]		1 [Reference]	
Provider referral	0.83 (0.76–0.91)	<.001	0.84 (0.74–0.95)	.01
**Insurance type**				
No insurance	1 [Reference]		1 [Reference]	
Medicaid	0.87 (0.78–0.97)	.01	0.68 (0.58–0.80)	<.001
Private insurance	0.97 (0.89–1.06)	.54	0.87 (0.77–0.99)	.04
**Social support**				
Good, very good, or excellent	1 [Reference]		1 [Reference]	
Poor or fair	1.17 (1.08–1.28)	.01	1.06 (0.94–1.20)	.36
**Other smokers in the home**				
No	1 [Reference]		1 [Reference]	
Yes	0.90 (0.84–0.97)	.01	0.83 (0.74–0.92)	.01
**Fagerström Test for Nicotine Dependence**	1.04 (1.03–1.06)	<.001	1.03 (1.00–1.05)	.02
**Mental health condition**				
No	1 [Reference]		1 [Reference]	
Yes	1.49 (1.38–1.61)	<.001	1.29 (1.15–1.44)	<.001
**Chronic health condition**				
No	1 [Reference]		1 [Reference]	
Yes	1.12 (1.03–1.21)	.01	1.14 (1.02–1.28)	.02
**Confidence to quit for 24 hours**				
Somewhat or not	1 [Reference]		1 [Reference]	
Very or extremely	1.12 (1.01–1.24)	.04	1.12 (0.96–1.30)	.14
**Postbaseline**				
**30-Day cessation at 7-month follow-up**				
Abstinent	—		1 [Reference]	
Not abstinent			2.89 (2.54–3.30)	<.001
**Number of telephone counseling sessions before 7-month follow-up**				
0–4	—		1 [Reference]	
≥5			0.94 (0.84–1.05)	.26
**Use of nicotine replacement therapy**				
No	—		1 [Reference]	
Yes			1.14 (1.00–1.29)	.05

Abbreviations: CI, confidence interval; OR, odds ratio.

a Model 1 included only baseline covariates for clients with complete data. *C* index = 0.60. *C* index measures goodness of fit; values range from 0.5 to 1.0; a *C* index of 0.5 indicates a model with no predictive ability.

b Model 2 included both baseline and follow-up covariates for clients with complete data. *C* index = 0.65. *C* index measures goodness of fit; values range from 0.5 to 1.0; a *C* index of 0.5 indicates a model with no predictive ability.

Secondary analyses showed that among clients who were not abstinent in the previous 30 days at 7-month follow-up (model 3), men, clients with Medicaid or private insurance, and clients who lived with other smokers were less likely to re-enroll. As in model 2, odds of re-enrollment in model 3 were higher among clients with a mental health condition or a chronic health condition than among those who did not have these conditions (Table 3).

**Table 3:** Likelihood of Re-Enrollment (Enrolling ≥2 Times) in the Arizona Smokers’ Helpline, Compared With One-Time–Only Enrollment, Among Clients Not Abstinent From Tobacco in Previous 30 Days at 7 Months After First Enrollment, January 1, 2011–June 26, 2016

Variable	Model 3 (n = 7,593)^a^	
	OR (95% CI)	*P* Value
**Baseline**		
**Age, per 10-year increase**	1.03 (0.98–1.09)	.20
**Sex**		
Female	1 [Reference]	
Male	0.78 (0.69–0.88)	<.001
**Mode of entry into program**		
Self-referral	1 [Reference]	
Provider referral	0.83 (0.72–0.95)	.01
**Insurance type**		
No insurance	1 [Reference]	
Medicaid	0.64 (0.53–0.77)	<.001
Private insurance	0.85 (0.74–0.98)	.03
**Social support**		
Good, very good, or excellent	1 [Reference]	
Poor or fair	1.08 (0.94–1.23)	.29
**Other smokers in the home**		
No	1 [Reference]	
Yes	0.81 (0.72–0.91)	.01
**Fagerström Test for Nicotine Dependence**	1.02 (0.99–1.04)	.18
**Mental health condition**		
No	1 [Reference]	
Yes	1.26 (1.11–1.42)	.01
**Chronic health condition**		
No	1 [Reference]	
Yes	1.19 (1.05–1.36)	.01
**Confidence to quit for 24 hours**		
Somewhat or not	1 [Reference]	
Very or extremely	1.08 (0.92–1.28)	.34
**Postbaseline**		
**No. of telephone counseling sessions before 7-month follow-up**		
0–4	1 [Reference]	
≥5	0.91 (0.80–1.04)	.17
**Use of nicotine replacement therapy**		
No	1 [Reference]	
Yes	1.11 (0.97–1.28)	.14

Abbreviations: CI, confidence interval; OR, odds ratio.

a *C* index = 0.58. *C* index measures goodness of fit; values range from 0.5 to 1.0; a *C* index of 0.5 indicates a model with no predictive ability.

## Discussion

Although previous research investigated strategies to re-engage clients in tobacco-cessation programs (ie, quitlines), to our knowledge, ours is the first study to investigate predictors of re-enrollment. Tailored strategies have been identified as a key element of interventions to recruit smokers into a cessation program (15). By examining the characteristics of former clients who re-engage in services, treatment programs such as quitlines can tailor programs and outreach strategies that target those at a high risk of relapse and who have greater odds of re-enrolling. We demonstrated that clients who reported using tobacco in the 30 days before 7-month follow-up, compared with clients who reported abstinence, were more likely to re-enroll in services. Men, clients referred to the quitline by their health care provider, and clients living with other smokers were less likely to re-enroll than women, self-referred clients, and clients not living with other smokers. Additionally, odds of re-enrollment among those who were not 30-day abstinent at 7-month follow-up were significantly higher for those who had a mental health and/or chronic health condition and those who used smoking-cessation medication during their first enrollment.

People who smoke require several quit attempts before they sustain tobacco abstinence (4). In our study, 62.6% of clients reported using tobacco in the 30 days before the 7-month follow-up survey; however, only 18.6% re-enrolled in services. Relapsed smokers, although interested in cessation services, are less likely to proactively seek treatment programs (6,16). This reluctance may be due to low levels of self-efficacy or confidence in the behavior-change process, which can adversely affect treatment-seeking behavior (17). Quitlines may be missing an opportunity to connect relapsed smokers to evidence-based cessation support. It is feasible to proactively re-engage relapsed callers by using techniques such as interactive voice response (IVR) and short message services (SMS, ie, text messages) (11); however, more research is warranted in this area.

Clients reporting a mental health condition and those with greater tobacco dependence were more likely to re-enroll than clients not reporting a mental condition and those with less tobacco dependence. Greater tobacco dependence and the presence of a mental health condition are barriers to successful abstinence (18,19). Our findings support the literature on high rates of smoking and nicotine dependence among populations with mental health conditions (20). Some strategies that quitlines can use to further re-engage clients with mental health conditions could include increasing frequency of contact using multimodal strategies (eg, email, text, telephone) after completion of treatment. These strategies would allow quitlines to check in with clients about their smoking progress and engage in proactive outreach efforts if clients report a relapse or barriers to staying quit. Bidirectional exchange or e-referrals between quitlines and health care providers is another potentially effective re-engagement strategy. Unlike traditional fax referrals, bidirectional e-referrals are sent from health care providers (based on the electronic health record) to the quitline, and quitlines can report back outcomes of service engagement to the electronic health record in an efficient closed loop. Such bidirectional exchange can support continuity of care and assist mental health providers by ensuring that they have information on clients who have engaged in quitline services. In particular, clients who relapse after treatment or who leave treatment without having quit can be re-referred to the quitline for re-engagement and support for further quit attempts.

Other factors that reduced the odds of re-enrollment in our study were the presence of other smokers in the home and having Medicaid insurance. Living with other smokers is a known barrier to quitting (21), and our results suggest that the social environment of smoking in the home can also impede treatment-seeking behavior. Quitlines may be in a unique position to identify the client’s social network and develop tailored re-engagement strategies (eg, proactive calls, retention/re-engagement mailings) for clients residing with other smokers or develop peer-support–based programs that include other smokers in the home. Furthermore, clients reporting low levels of social support for quitting were more likely to re-enroll in quitline services, which in part could have resulted from the social support and skills-building components inherent in the telephone counseling sessions. The proactive counseling process promotes accountability and building of social support. For those lacking social support, re-enrollment in the quitline may be beneficial.

Differences between men and women in quitline service use is an emerging area of research, and research shows that such differences exist in quit outcomes (22). Our study is the first to find that men are less likely than women to re-engage in cessation services, consistent with evidence showing that men in general underuse quitlines (23). This finding may indicate the need for tailored outreach and re-engagement strategies for male smokers (eg, use of multimodal strategies such as apps and text messages to increase adherence and re-engagement). Finally, clients who were referred by their health care providers were less likely than self-referred clients to re-engage in services. Although patient caseload and health care system strain may result in a lack of standardization in provider referral systems and inconsistent referrals sent to quitlines (24), providers are credible sources of behavior-change advice for smokers. Provider-referral systems can increase access to tobacco cessation services, including directing former clients to re-engage in cessation services.

The strengths of our study were a large sample of quitline clients and all clients having access to the standardized protocols for engagement in services. Our study had several limitations. One is the use of a single-item yes-or-no measure to assess mental health, which precluded our ability to assess differences in client re-engagement by type of mental health condition. Second, we were unable to obtain data on duration of use of smoking-cessation medication, adherence to medication dosage, and reasons for lack of adherence. Because our study was retrospective, it was subject to selection bias, recall bias, and missing data, which may reduce the generalizability of study results. However, the only data that were truly retrospective were the data on 30-day tobacco cessation and use of NRT (collected at 7-month follow-up); all other data were collected at baseline (prospectively). Furthermore, we fit 2 models, one of which included only baseline variables and therefore had less missing data. The results were similar between the 2 models, indicating that missing data are not likely to be a problem in the interpretation of the results. Finally, 30-day quit status was a self-reported measure. However, the collection of self-reported data is common practice among quitlines (25).

Because nicotine dependence is chronic and relapsing, quitlines may be missing an opportunity to re-engage former clients who may be at high risk of relapse. Our results showed that clients who were not tobacco abstinent at the end of their first enrollment were more likely than clients who were tobacco abstinent to re-engage in quitline services. Furthermore, among those who were not abstinent, only 10% re-enrolled in services. Understanding factors that predict client re-enrollment in smoking-cessation services can help quitlines tailor strategies to proactively re-engage high-risk clients to promote long-term cessation outcomes.
